# Stressful Life Events and Risk of Depression 25 Years Later: Race and Gender Differences

**DOI:** 10.3389/fpubh.2016.00049

**Published:** 2016-03-24

**Authors:** Shervin Assari, Maryam Moghani Lankarani

**Affiliations:** ^1^Department of Psychiatry, University of Michigan, Ann Arbor, MI, USA; ^2^Center for Research on Ethnicity, Culture and Health (CRECH), School of Public Health, University of Michigan, Ann Arbor, MI, USA; ^3^Medicine and Health Promotion Institute, Tehran, Iran

**Keywords:** race, sex, gender, population differences, depression, depressive disorder, psychological stress

## Abstract

**Background:**

Although stressful life events (SLEs) predict subsequent risk of developing a major depressive episode (MDE), limited information exists on whether or not race and gender alters the predictive role of SLE on risk of MDE over a long-term period. The current study explored race and gender differences in the long-term predictive role of SLE at baseline (1986) on subsequent risk of MDE 25 years later (2011) in a nationally representative cohort in the United States.

**Methods:**

Using a life course epidemiological approach, this longitudinal study borrowed data from the Americans’ Changing Lives (ACL) Study 1986–2011. Main predictor of interest was baseline SLE over the last 3 years measured at 1986. Main outcome was risk of MDE [Composite International Diagnostic Interview (CIDI)] 25 years later (2011). Covariates included demographics, socioeconomics, depressive symptoms [Center for Epidemiological Studies-Depression Scale (CES-D)], chronic medical conditions, and health behaviors measured at baseline (1986). Gender and race were the focal moderators. We employed logistic regressions in the pooled sample, and specific to race and gender, to test whether or not SLE × race and SLE × gender interactions are significant.

**Results:**

In the pooled sample, baseline SLE (1986) predicted risk of MDE 25 years later (2011). We also found a gender by SLE interaction, suggesting a stronger predictive role of SLE for subsequent clinical depression for men compared to women. Race did not modify the predictive role of SLE on subsequent risk of MDE 25 years later.

**Conclusion:**

How SLE predicts MDE 25 years later differs for men and women, with a stronger predictive role for men compared to women. More research is needed to better understand the complex links between gender, sex, stress, and depression.

## Introduction

Regardless of the type of stressor (1, 2), literature has consistently shown a strong association between stressful life events (SLEs) and the risk of depression (3). As a result, researchers have used a number of stressors (i.e., SLE) (4) as an indicator of depression risk (1, 2). The role of SLE as a major risk factor for depression in clinical and community settings has been confirmed by meta-analyses and systematic reviews (3, 5–7). However, most of our knowledge is on the role of SLE as a predictor of risk of depression over a short period of time (8), which is a trigger (9) rather than SLE as a predictor of the long-term risk of depression (9). More is yet to be learned about the predictive role of SLE on the long-term risk of depression (9). In this view, SLE is being conceptualized as a proxy of the life environment of the individual, rather than a trigger of a depressive episode in the near future. This is in line with a life course perspective that suggests early exposures will have long-term effects over the life course (10–14).

There is a debate regarding whether or not stress response is independent of setting and context (5, 15, 16), or is context dependent (17–21). Despite decades of research to understand characteristics that alter the effect of stress on depression (22, 23), our information is still limited on factors that mitigate the SLE–depression association (24–27). As the effects of SLE largely should depend on characteristics of the individual who experiences stress, and also the context in which stress occurs (28), it is plausible to hypothesize that the effect of SLE on MDD may vary for populations (29, 30). In line with this *differential effect* hypothesis, sub-populations endorse different levels of vulnerability to the effect of risk and protective factors (31). In this view, all associations in health including the SLE–depression association are a heterogenic relation, varying from one population to another (31). This is in line with a growing body of evidence on population variation in the effects of other risk and protective factors (12–14, 25, 27, 32–36).

Race may alter the link between SLE and depression at each time point, and also over time. Despite higher levels of exposure to SLE (37), Blacks have disproportionally lower rates of depression (38), a phenomenon called the Black–White paradox (39, 40). Thus, race alters how stress is associated with risk of depression (41). In a recent study in 2015, Assari used data from the National Survey of American Life (NSAL), 2003, a cross-sectional survey of 5008 Blacks, and 891 Non-Hispanic Whites and showed that among men, the SLE × race interaction was significant, suggesting a stronger association between SLE and major depressive episode (MDE) among White men compared to Black men. Such interaction between SLE × race was not found among women. Authors concluded that the association between SLE and depression may be stronger for White men than Black men; however, this link does not differ between White and Black women (27). In another study by Moazen, Zadeh, and Assari, psychological distress measured by number of depressive symptoms in 1986, which predicted MDD 15 years later for Whites failed to predict MDD for Blacks. The authors concluded that psychological measures that count subjective symptoms may fail to have more objective measures, such as MDD in the future among Blacks (14). None of these studies have, however, focused on the long-term predictive role of SLE on subsequent risk of MDD.

Gender is also another main factor that may alter the cross-sectional and long-term associations between experiences of stress and the risk of MDD. Based on the psychosocial theory of depression, predominance of MDD in women is at least in part mediated by a higher exposure of women to SLE during childhood and adulthood (42, 43). Another main hypothesis for higher prevalence of depression among women is gendered response to SLE (44). Biological mechanisms, such as hormones may also be involved in role of sex as a moderator of the effect of stress on depression (45–47). In this study, however, we conceptualized gender as a social construct that shapes life experiences, exposures, and resources, and coping styles (48, 49).

We conducted this study to explore race and gender differences in the predictive role of SLE in 1986 on long-term risk of depression 25 years later in 2011. Similar to previous work (25–27), we conceptualized race and gender as potential moderators of the stress–MDE link. This study focuses on the contextual effects of race and gender as moderators of the effects of risk factors rather than the main effects of gender or race on outcome above and beyond risk factors. To provide results generalizable to the United States, we used nationally representative data.

## Materials and Methods

### Design and Setting

Data were from wave 1 (1986) and wave 5 (2011) of the Americans’ Changing Lives (ACL) Study, a nationally representative longitudinal study in the United States (50, 51). Appendix 1 in Supplementary Material provides additional information on sampling, follow-up, and data collection.

### Original Study

The ACL study is the oldest ongoing nationally representative longitudinal study on the role of a broad range of psychosocial factors on health changes with aging over the life course from adulthood to early elderly (52).

### Ethics

The original study received approval from the institutional review board (IRB), University of Michigan. Informed consent was obtained from all individual participants included in the study. All procedures performed in studies involving human participants were in accordance with the ethical standards of the institutional and/or national research committee and with the 1964 Helsinki declaration and its later amendments or comparable ethical standards.

### Participants and Sampling

The ACL used a stratified multistage probability sampling strategy. The ACL has oversampled African Americans and older adults (age 60 and above). In 1986, the study enrolled 3,617 community-dwelling adults age 25 or older who lived in the continental U.S. Wave 1 included 70% of sampled households and 68% of sampled individuals. Wave 5 included 81% of survivors in 2011. More information on participants, sampling, and recruitment is shown in Appendix 1 in Supplementary Material (52, 53).

### Analytical Sample

The current analysis is limited to 1,129 individuals who were followed for 25 years. The study is on the role of SLE in 1986 on MDE 25 years later. So, only survivors and those who participated in wave 1 and wave 5 could enter this study.

### Process

First, wave data were collected via face-to-face interviews. Wave 5 data were collected via either face-to-face or telephone interviews. Telephone interviews are accepted for Composite International Diagnostic (54) and other structured diagnostic interviews (55).

### Measures

Baseline data were collected on demographic characteristics, socioeconomic status, health behaviors, depressive symptoms, and health at wave 1 (1986). MDE was measured at wave 5 (2011).

### Stressful Life Events

We collected data on the number of major negative events in the past 3 years. Participants were asked about SLE at wave 1 (1986), using a measure that accords well with current standards of measurement of major/traumatic events (56). Although more comprehensive stress measures have been developed since 1986 (57), there is still no gold standard for measurement of stress (57).

### Demographics

Demographic variables included age (continuous measure), race (Black and White, with White as the reference group), and gender (dichotomous variable with male as the reference group). While age was a covariate, race and gender were moderators.

### Socioeconomics

Socioeconomic status was measured using education (years of schooling) and income [an 11 – level categorical (rank) variable treated as a continuous measure].

### Chronic Medical Conditions

We collected data on chronic medical conditions (CMC) by measuring self-reported data. Participants were asked whether or not health care providers have ever told them that they had any of the following seven conditions: hypertension, diabetes, chronic lung disease, heart disease, stroke, arthritis, and cancer. Participants were also asked if they were currently taking medication for these conditions. Based on dichotomous responses, we calculated a sum score, ranging from 0 to 7, with a higher score indicating more CMC (51).

### Self-Rated Health

Respondents were asked to classify their self-rated health (SRH) as excellent, very good, good, fair, or poor. SRH was operationalized in the following two ways: (1) as a dichotomous measure and (2) as a continuous score. For the first approach, we collapsed this five-category scale into two categories (fair/poor vs. excellent/very good/good), a cutoff point that is common in the literature. This measure has shown high test–retest reliability and validity, when considering its predictive power for mortality and other health outcomes (58, 59) (1 = excellent and 5 = poor).

### Function

We collected information on respondents’ functional health fusing several questions. A score of 1 represents confinement to a bed or chair and a score of 4 represents the ability to do heavy work inside or outside of the home. These scores were then transformed into a three-category variable: (1) no functional limitation (i.e., able to do heavy work around the house); (2) some limitation, meaning the respondent reported not being able to do such things as heavy physical labor or work around the house; or (3) moderate/severe limitation, meaning the respondent reported having great difficulty walking a few blocks or climbing stairs, or reported being confined to a bed or a chair (57).

### Obesity

Obesity was defined based on the body mass index (BMI) of larger than 30 kg/m^2^. The BMI level was calculated based on self-reported weights and heights. Weight and height were originally collected in pounds (1 pound = 0.453 kg) and feet (1 foot = 0.3048 m)/inches (1 inch = 0.0254 m), respectively. BMI calculated based on self-reported weight and height is known to be closely correlated with BMI based on direct measures of height and weight (60).

### Health Behaviors

The study also used ACL measures on exercise (physical activity), smoking (i.e., tobacco use), and drinking (i.e., alcohol consumption). The first measure, the physical activity index, asked respondents how often they engaged in the following activities: working in the garden or yard, participating in active sports or exercise, and taking walks. A 4-point Likert scale response ranged from “often” to “never.” The index was scored by taking the mean of the three items (61). A high value scored by respondents indicated a high level of physical activity. To measure smoking behavior, we asked respondents whether they currently smoke. A dummy variable was created where 1 = current smoker and 0 = non-smoker. A similar dummy measure was used concerning alcohol use, that is, whether or not the respondent currently drinks (1 = current drinker and 0 = non-drinker) (62).

### Depressive Symptoms

Depressive symptoms were measured with a brief version of the Center for Epidemiological Studies-Depression scale (CES-D) which included 11 items (63). Items measured the extent to which in the past week respondents felt depressed, happy, lonely, sad, restless sleep, that everything was an effort, that people were unfriendly, that they did not feel like eating, that people dislike them, that they could not get going, and that they enjoyed life. Item responses were 1 (“hardly ever”) to 3 (“most of the time”). Positively worded items were reverse-coded, and a mean score was computed across all 11 items (32, 64, 65), resulting in a continuous measure of depressive symptoms for baseline, ranging from 1 to 3. Higher scores indicated more severe depressive symptoms. This abbreviated CES-D has acceptable reliability and a similar factor structure to the original version (33).

### Cognitive Impairment

The ACL survey has adopted the five-items of the Short Portable Mental Status Questionnaire (SPMSQ) to measure the cognitive impairment of the respondents. The SPMSQ assesses respondents’ memory, knowledge of current events, and ability to perform mathematical tasks and is designed to identify cognitive deficits among community and institutionalized settings (66, 67). The measure used the following four items: (a) “What is the date today – month, day, and year?” (b) “What day of the week is it?” (c) “What is the name of the president of the United States?” (d) “Subtract 3 from 20 and tell me the number you get. Then, keep subtracting 3 from this number and each new number you get, telling me the results as you go (Stop when the answer is 2 or less).” Each item was coded as 0 “correct” or 1 “incorrect,” with a total score representing the cognitive impairment (higher scores indicate poorer cognitive functioning) (68).

### Clinical Depression

The outcome variable was the 12-month MDE measured at 2011 using the World Mental Health Composite International Diagnostic Interview (CIDI). CIDI is a fully structured diagnostic interview and evaluates a wide range of Diagnostic and Statistical Manual-IV (DSM-IV) psychiatric disorders, including but not limited to MDE. CIDI has been used reliably on the World Mental Health project (64).

### Statistical Analysis

Due to the complex sample design used in the HRS, Stata 13.0 (Stata Corp., College Station, TX, USA) was used for data analysis. Taylor series linearization was used for estimation of SEs. Thus, the stratified and clustered data, as well as non-response patterns, were considered for data analysis. Using weights enabled us to provide rates that are generalizable to the U.S. population.

For univariate analyses, we reported means or frequencies (%) when appropriate. For multivariate analysis, we used logistic regression models in the pooled sample, and also stratified by race and gender. SLE was the main predictor of interest, and the outcome was risk of endorsement for MDE measured in 2011 using CIDI. Covariates included baseline demographics, socioeconomics, depressive symptoms (CES-D), physical health (hypertension, diabetes, chronic lung disease, heart disease, stroke, cancer, and arthritis), and health behaviors measured in 1986. Gender and race were the focal moderators.

We did not control for SLE in 2011. Controlling for SLE concurrent with outcome is needed when the focus is to know the residual effects of SLE in 1986 on MDE in 2011 net of SLE in 2011. Thus, the goal of this study is to study predictive (not residual) effect of SLE on MDE. With that goal, controlling for SLE in 2011 will be a case of over-adjustment, as higher SLE in 2011 may be in the causal path for the effect of SLE in 1986 on MDE in 2011. A significance level of *p* < 0.05 was considered as statistically significant. Adjusted odds ratios (OR) with 95% confidence intervals (CI) are reported.

## Results

The current analysis included 1,129 individuals who were followed for 25 years and completed surveys in wave 1 and wave 5 of the ACL study. Participants were White men (*n* = 294), Black men (*n* = 108), White women (*n* = 490), and Black women (*n* = 237).

Table 1 provides descriptive statistics for age, education, income, depressive symptoms, CMC, and health behaviors at baseline by race and gender. The majority of participants (64%) were females with a mean age of 41 (SD = 11) years at baseline (Table 1).

**Table 1:** **Descriptive statistics overall and based on race and gender**.

	Mean (SD)	Min–Max	Mean (SD)	Min–Max	Mean (SD)	Min–Max	Mean (SD)	Min–Max	Mean (SD)	Min–Max
					
	All		Whites		Blacks		Men		Women	
Age	47.77 (0.53)	46.69–48.84	47.96 (0.60)	46.75–49.17	46.33 (0.72)	44.89–47.78	46.44 (0.66)	45.10–47.78	48.96 (0.59)	47.77–50.15
Education^a^	12.53 (0.10)	12.34–12.73	12.69 (0.11)	12.48–12.90	11.37 (0.23)	10.90–11.84	12.74 (0.13)	12.47–13.00	12.35 (0.09)	12.18–12.53
Income^a^	5.41 (0.09)	5.22–5.60	5.57 (0.10)	5.36–5.77	4.25 (0.18)	3.88–4.62	5.78 (0.11)	5.57–6.00	5.08 (0.10)	4.88–5.27
CMC^a^	0.79 (0.03)	0.74–0.85	0.78 (0.03)	0.71–0.84	0.91 (0.05)	0.81–1.02	0.68 (0.04)	0.61–0.76	0.89 (0.03)	0.83–0.96
Function^a^	3.72 (0.02)	3.69–3.75	3.72 (0.02)	3.69–3.76	3.69 (0.03)	3.62–3.76	3.78 (0.02)	3.73–3.82	3.67 (0.02)	3.63–3.71
Cognition^a^	0.69 (0.03)	0.62–0.76	0.65 (0.04)	0.57–0.73	1.00 (0.05)	0.90–1.10	0.67 (0.04)	0.60–0.74	0.72 (0.04)	0.63–0.80
SLE^a^	0.88 (0.02)	0.84–0.92	0.88 (0.02)	0.84–0.92	0.87 (0.03)	0.81–0.94	0.85 (0.03)	0.79–0.91	0.90 (0.02)	0.86–0.95

*^a^*p* < 0.05 for comparison of Blacks and Whites*.

Table 2 provides a summary of four logistic regressions in the pooled sample. Gender interacted with baseline SLE on the risk of MDE 25 years later, suggesting a stronger predictive role of SLE on the risk of subsequent clinical depression for men compared to women. Race did not modify the SLE–MDE association (Table 2).

**Table 2:** **Summary of logistic regressions in the pooled sample with and without interactions**.

	OR (SE)	95% CI	OR (SE)	95% CI	OR (SE)	95% CI	OR (SE)	95% CI
				
	Model 1 Main effects		Model 2 Main effects + gender interaction		Model 3 Main effects + race interaction		Model 4 Main effects + both interactions	
Gender (females)	1.12 (0.28)	0.68–1.84	2.38 (1.28)	0.81–7.03	1.12 (0.28)	0.68–1.84	2.39 (1.29)	0.81–7.09
Race (blacks)	0.92 (0.23)	0.55–1.54	0.88 (0.22)	0.54–1.45	1.04 (0.51)	0.39–2.77	1.05 (0.50)	0.41–2.72
Age	0.98 (0.01)	0.96–1.01	0.98 (0.01)	0.96–1.01	0.98 (0.01)	0.96–1.01	0.98 (0.01)	0.96–1.01
Education	0.97 (0.08)	0.83–1.13	0.97 (0.07)	0.83–1.13	0.97 (0.08)	0.83–1.13	0.97 (0.07)	0.83–1.13
Income	0.98 (0.06)	0.86–1.11	0.98 (0.06)	0.88–1.10	0.98 (0.06)	0.86–1.11	0.98 (0.06)	0.88–1.10
Chronic medical conditions	1.18 (0.20)	0.85–1.65	1.15 (0.19)	0.82–1.61	1.18 (0.20)	0.85–1.66	1.15 (0.19)	0.82–1.61
Self-rated health (Poor)	1.37 (0.79)	0.43–4.40	1.46 (0.80)	0.49–4.40	1.37 (0.79)	0.43–4.41	1.47 (0.80)	0.49–4.40
Obese	1.36 (0.36)	0.80–2.30	1.31 (0.38)	0.73–2.35	1.36 (0.36)	0.80–2.30	1.31 (0.38)	0.73–2.36
Function	0.86 (0.25)	0.48–1.55	0.84 (0.23)	0.48–1.46	0.86 (0.25)	0.48–1.55	0.84 (0.23)	0.48–1.46
Cognition	0.94 (0.12)	0.73–1.20	0.94 (0.11)	0.74–1.20	0.94 (0.12)	0.73–1.21	0.95 (0.11)	0.75–1.20
Depressive symptoms (CES-D)	2.58 (0.85)*	1.33–4.99	2.73 (0.83)**	1.47–5.05	2.58 (0.85)*	1.33–5.00	2.72 (0.84)*	1.47–5.05
Smoking	1.61 (0.45)	0.91–2.84	1.63 (0.46)^#^	0.92–2.89	1.60 (0.45)	0.91–2.80	1.62 (0.45)^#^	0.92–2.85
Drink	0.74 (0.16)	0.48–1.13	0.74 (0.16)	0.47–1.14	0.74 (0.16)	0.48–1.13	0.73 (0.16)	0.47–1.14
SLE	1.41 (0.22)*	1.03–1.93	2.09 (0.60)*	1.17–3.74	1.58 (0.71)	0.64–3.92	2.46 (1.37)	0.80–7.55
SLE × gender (females)			0.54 (0.16)*	0.29–1.00			0.53 (0.17)*	0.28–1.00
SLE × race (blacks)					0.90 (0.26)	0.51–1.61	0.87 (0.25)	0.49–1.54

*SLE, stressful life events; CES-D, Center for Epidemiological Studies-Depression Scale*.

*^#^*p* < 0.1*.

*p < 0.05.

**p < 0.01.

***p < 0.001.

Table 3 provides a summary of logistic regressions based on race and gender, with and without SES in the model. Neither race nor gender had main effects on risk of MDD while SLE was controlled. SLE was associated with risk of MDD among men and women. However, SLE was associated with risk of MDD among Whites but not Blacks. These associations stayed in the model when SES was introduced to the model (Table 3).

**Table 3:** **Summary of logistic regressions based on race and gender**.

	Men		Women		Whites		Blacks	
	OR (SE)	95% CI	OR (SE)	95% CI	OR (SE)	95% CI	OR (SE)	95% CI
**Model 1**								
Gender (Females)	–	–	–	–	1.47 (0.44)	0.80–2.67	1.20 (0.52)	0.49–2.91
Race (Blacks)	1.29 (0.59)	0.51–3.24	1.20 (0.39)	0.63–2.30	–	–	–	–
Age	0.97 (0.02)	0.92–1.01	0.99 (0.01)	0.96–1.02	0.98 (0.01)	0.96–1.01	0.97 (0.02)	0.93–1.03
SLE	2.10 (0.58)*	1.20–3.66	1.31 (0.19)^#^	0.98–1.74	1.60 (0.30)*	1.10–2.32	1.30 (0.22)	0.93–1.82
**Model 2**								
Gender (females)	–	–	–	0.46–1.99	1.37 (0.39)	0.77–2.44	1.07 (0.50)	0.42–2.75
Race (Blacks)	1.31 (0.54)	0.57–2.99	0.96 (0.35)	–	–	–		
Age	0.97 (0.02)	0.93–1.01	0.99 (0.01)	0.96–1.02	0.98 (0.01)	0.96–1.01	0.97 (0.02)	0.93–1.01
Education	0.97 (0.13)	0.74–1.27	0.87 (0.08)	0.73–1.03	0.92 (0.08)	0.76–1.10	0.88 (0.12)	0.66–1.17
Income	0.93 (0.12)	0.72–1.21	0.94 (0.07)	0.81–1.09	0.92 (0.06)	0.80–1.06	0.98 (0.08)	0.83–1.16
SLE	2.09 (0.57)*	1.20–3.63	1.26 (0.18)	0.93–1.69	1.52 (0.29)*	1.05–2.22	1.34 (0.24)	0.94–1.92

*SLE, stressful life events; CES-D, Center for Epidemiological Studies-Depression Scale*.

*^#^*p* < 0.1*.

*p < 0.05.

**p < 0.01.

***p < 0.001.

Tables 3 and 4 provide a summary of logistic regressions based on race and gender. While baseline SES, health, depressive symptoms, and health behaviors were in the model, SLE only predicted MDD among men but not women. We also found that SLE only predicted MDD among Whites but not Blacks.

**Table 4:** **Summary of full logistic regressions based on race and gender**.

	OR (SE)	95% CI	OR (SE)	95% CI	OR (SE)	95% CI	OR (SE)	95% CI
				
	Men		Women		Whites		Blacks	
Gender (females)	–	–	–	–	1.13 (0.32)	0.64–1.98	0.92 (0.47)	0.33–2.58
Race (Blacks)	1.17 (0.49)	0.50–2.73	0.73 (0.27)	0.35–1.54	–	–	–	–
Age	0.95 (0.02)*	0.91–1.00	1.00 (0.02)	0.96–1.04	0.98 (0.01)	0.95–1.01	0.96 (0.02)	0.92–1.01
Education	1.00 (0.14)	0.76–1.33	0.93 (0.08)	0.77–1.11	0.97 (0.09)	0.80–1.17	1.00 (0.12)	0.79–1.27
Income	0.99 (0.12)	0.77–1.27	0.99 (0.07)	0.87–1.14	0.97 (0.07)	0.84–1.12	1.02 (0.08)	0.87–1.19
Chronic Medical Conditions	1.95 (0.57)*	1.09–3.51	0.81 (0.17)	0.52–1.24	1.19 (0.23)	0.81–1.76	1.07 (0.37)	0.54–2.13
Self-rated health (Poor)	1.39 (1.75)	0.11–17.52	1.57 (0.83)	0.54–4.53	1.45 (0.98)	0.37–5.70	1.09 (0.68)	0.31–3.85
Obese	0.83 (0.52)	0.24–2.91	1.81 (0.52)*	1.01–3.24	1.29 (0.41)	0.68–2.45	1.90 (0.88)	0.74–4.86
Function	1.11 (1.00)	0.18–6.79	0.78 (0.20)	0.46–1.32	0.79 (0.26)	0.40–1.54	1.22 (0.33)	0.71–2.11
Cognition	0.90 (0.19)	0.58–1.38	0.98 (0.13)	0.75–1.28	0.83 (0.14)	0.59–1.17	1.63 (0.34)*	1.07–2.49
Depressive Symptoms (CES-D)	1.80 (0.78)	0.75–4.29	3.49 (1.23)***	1.72–7.08	2.45 (0.94)*	1.13–5.31	3.42 (1.31)**	1.58–7.40
Smoking	1.62 (0.59)	0.78–3.37	1.60 (0.53)	0.82–3.11	1.51 (0.47)	0.81–2.84	1.93 (0.67)^#^	0.96–3.90
Drink	0.93 (0.50)	0.32–2.72	0.69 (0.21)	0.38–1.26	0.67 (0.15)^#^	0.42–1.07	1.67 (0.72)	0.69–4.00
SLE	1.94 (0.49)*	1.17–3.24	1.15 (0.15)	0.87–1.50	1.43 (0.27)^#^	0.98–2.08	1.29 (0.21)	0.93–1.79

*SLE, stressful life events; CES-D, Center for Epidemiological Studies-Depression Scale*.

*^#^*p* < 0.1*.

*p < 0.05.

**p < 0.01.

***p < 0.001.

## Discussion

In line with the *differential effect hypothesis* (31, 34–36), gender influenced the predictive role of baseline SLE on the risk of MDE 25 years later, suggesting a stronger predictive role for men compared to women. We could not find racial differences in the predictive role of baseline SLE on subsequent risk of MDE.

Our findings may be due to gender differences in the threshold of reporting stress. Our findings may also be due to the gender differences in risk perception (69–71). These findings may also be explained by gender differences in use of internalizing or externalizing coping in response to stress (72). Women may have a more conservative threshold for perception or report of stressors. Similar to other socio-demographic factors, gender shapes availability, access, use, and efficacy of intra-personal and inter-personal resources and assets in response to stress (73). Men and women differently handle stress that influences resilience (73).

Race and gender also determine the risk of exposure to stress (74–76). Differential exposure to stress over the life course will influence susceptibility to stressors later in life. Black men and women experience high levels of exposure of racism in their daily lives (74), and Black women also experience sexism as a major stressor (74, 75). Differential vulnerability to stress across groups may be in part due to differential exposure to stress over the life course (77, 78). Such differential vulnerability has been shown by differential cortisol profile based on race (79, 80). For instance, in a study, Black women showed greater stress reactivity than White women. More Blacks (58%) than Whites (14%) showed cortisol reactivity from rest to 15-min post-challenge (79). Blacks also have lower levels of wake-up cortisol and less steep early and late daily cortisol decline relative to Whites (80). Despite these race differences having implications for the role of SLE on depression, we did not find any race difference in such a link.

Gender and race may change availability, access, use, and effects of stress buffers, such as family (81) and religion (73). Regarding gender, women are better users of religiosity and social support in coping with stress (82, 83). Women more commonly use faith and family to deal with stress (84–86) and seek resilience through God, faith, and social support (84–89).

Literature has suggested that race, gender, and their interstations have implications for coping styles, including but not limited to use of problem-focused or emotional coping styles (90). It has been shown that older Black men may have higher tendency to implement adaptive coping strategies, including positive reappraisal and maintenance of hope and optimism (91), while Black women may tend toward religious coping, avoidant coping, wishful-thinking, seeking social support, and emotion expression (92, 93). Such race by gender differences in coping and cognition following stress have implications for resilience and stress reactivity (54).

Although our study conceptualized gender not sex as the moderator of the stress–depression link, our findings are supported by a literature on the role of sex as a moderator of the interactions between stress, genetic predisposition, and depression. For instance, Kurrikoff et al., who did not find a main effect of the 5-HTTLPR genotype or the interaction between 5-HTTLPR and SLE on MDD documented the 5-HTTLPR × gender interaction on MDD in case of interpersonal SLE on MDE. The study showed the lowest prevalence of depression among female s′-allele carriers who had low levels of exposure to interpersonal adverse events. Authors concluded that the complex interplay between serotonin, social stress, and depression is sex dependent (94). Other studies have also shown sex differences in the links between 5-HTTLPR s-allele and mental health response to stressors (95, 96). In the study by Kurrikoff et al., men had higher depressiveness in association with higher exposure to interpersonal adverse events if they had the l′/l′ genotype (94). Other studies have also shown that males with the l′/l′ genotype are more sensitive to SLE (96, 97).

Our study had its own limitations. Reliability and validity of SLE and depression measures may depend on race and gender (98). As our sample was limited to survivors of the ACL who were under follow up for 25 years, differential attrition based on race and gender may have biased the results. We cannot rule out that our results are not due to under-reporting of stress and depression among men. Despite the fact that SES, stress, physical health, and health behaviors are subject to change, we did not use them as time-varying covariates. In addition, life event checklists may not be the best way to measure life events (99). Mere measurement of recent life events may not adequately capture variation in stress exposure over the life course (100, 101). Despite their limitations (99, 100, 102), life event inventories are still the most commonly used instruments for the measurement of stress exposure (103, 104). Collecting rich contextual data on each SLE requires highly trained interviewers and is a labor-intensive task (28, 105).

Our study did not consider type of stressors. First and foremost, because current SLEs were not included in the model, and as we know that individuals with higher levels of SLEs at one time point are more likely to continue to experience SLEs, we cannot rule out the possibility that the effects of SLE on MD are not due to continuity across time in the level of SLEs. Thus, the results should not be interpreted as the residual effect of SLE in 1986 over SLE in 2011 due to lack of controlling for concurrent SLE at the time of outcome (2011). Second, men and women experience different types of SLEs, such as unemployment, child-care, domestic violence, and caregiving. Such gender differences may explain why men and women differ in the link between count of stressors and risk of depression (106). Men and women may also differ in their sensitivity to particular types of stress. For instance, while men are more likely to experience depressive episodes following stress related to work, divorce, and separation, women are more vulnerable to other SLE, such as conflict, serious illness, or death, in their proximal social network (107, 108). Women may be more vulnerable to SLE with an interpersonal nature (42, 109), such as romantic and marital relationships, childrearing, and parenting (109). Divorce, social support, and marital satisfaction may also differently link to depression among men and women (110). Men and women also differ in rumination of stressors and negative thoughts (82, 83, 111–116) that has implications for the contribution of SLE to the risk of psychopathology (116). In one study, gender differences in rumination, stress, and mastery mediated gender differences in depressive symptoms (115). All these studies support conceptualization of gender as an effect modifier for the stress – depression link (117).

Despite the above limitations, our study makes a unique contribution to the literature on race and gender differences on the psychological response to stress, and disparities in development of depression over a long period of time. Based on our findings, even in the presence of the same level of stress exposure, long-term mental health consequences of stress may depend on gender (118). However, this study cannot rule out the possibility of under-reporting SLE and MDE among men as a contributor to the gender differences found in this study.

The mechanism behind gendered response to stress is still unclear (119). Future research is needed to understand how contextual factors, such as gender and race, alter the long-term mental health effects of exposure to similar levels of stress. Such research will help us better understand racial and gender differences in epidemiology of depression as one of the most common and disabling psychopathologies (103, 120, 121). Researchers should not simply assume that populations are similarly vulnerable to social adversities. We believe that heterogenic vulnerability and resilience may be major mechanisms behind disparities in the epidemiology of psychiatric disorders across populations. Further research is needed on gender- and race-specific mediators and buffers of differential vulnerability, including personality, attribution styles, coping, social support, and religiosity.

To conclude, gender, but not race, altered the longitudinal association between the baseline level of SLE (1986) and risk of MDE 25 years later (2011). Thus, each incremental increase in SLE may better predict risk of MDE 25 years later among men compared to women. The predictive role of SLE for long-term risk of MDE seems to be similar for Whites and Blacks. Additional research is needed on the complex links between gender, race, exposure to stress, and development of depression.

## Research Involving Human Participants

Informed consent was obtained from all individual participants included in the study. All procedures performed in studies involving human participants were in accordance with the ethical standards of the institutional and/or national research committee and with the 1964 Helsinki declaration and its later amendments or comparable ethical standards.

## Author Contributions

SA designed the work and analyzed the data. ML drafted the manuscript and revised the draft. Both authors approved the final draft.

## Conflict of Interest Statement

SA and ML do not have any potential conflicts of interest to report.
