# Supplementary material for: Stressful Life Events and Risk of Depression 25 Years Later: Race and Gender Differences
Source: Front Public Health. 2016 Mar 24;4:49. doi: 10.3389/fpubh.2016.00049 (PMC4805579; doi:10.3389/fpubh.2016.00049)
Supplement: Supplementary file 1 [file Data_Sheet_1.DOCX]

**Appendix 1:** Additional information of sampling and follow up in Americans’ Changing Lives Study (ACL) **^1^**

| **Scope:** nationally representative longitudinal study  **Goal:** especial focus on differences between Black and White Americans in middle and late life.  **Initial Sample:**  For Wave I, a multistage stratified area probability sample with oversampling of Blacks and those aged 60 and older was used. For Wave II, an attempt was made to contact all respondents from Wave I (N = 3,617).  **Follow up Samples:** The number of respondents that participated in Wave II was 2,867. For Wave III, an attempt was made to contact all the respondents from Waves I and II. The number of respondents that participated in Wave III was 2,562. For Wave IV, an attempt was made to contact all respondents from Waves I, II, and III. The number of respondents that participated in Wave IV was 1,787. Finally, for Wave V, an attempt was made to contact all respondents from Waves I, II, III, and IV. The number of respondents that participated in Wave V was 1,427. In addition, some Wave III, IV, and V interviews were done by proxy respondents and not by the original Wave I respondents. [Wave III N=164; Wave IV N=95; Wave V N=108]  **Date of Collection:** 1986 1989 1994 2002 2011  **Data collection Mode:** Wave I of the study began in 1986 with a nation face-to-face survey of 3,617 adults ages 25 and up, with Black Americans and people aged 60 and over over-sampled at twice the rate of the others. Wave II constitutes face-to-face re-interviews in 1989 of those still alive. Survivors have been re-interviewed by telephone, and when necessary face-to-face, in 1994, 2001/02, and 2011, making up Waves III, IV, and V of the data. |
| --- |

1. House, JS. Americans' Changing Lives: Waves I, II, III, IV, and V, 1986, 1989, 1994, 2002, and 2011. ICPSR04690-v7. Ann Arbor, MI: Inter-university Consortium for Political and Social Research [distributor], 2014-09-09. http://doi.org/10.3886/ICPSR04690.v7
